# Chill or Thrill? The Effect of Storage Temperature Regime on *Listeria* Growth in Fresh-Cut Fruit Cocktails

**DOI:** 10.3390/foods14203523

**Published:** 2025-10-16

**Authors:** Beatrix W. Alsanius, Sofia Windstam, Emina Mulaosmanovic

**Affiliations:** Subject Area Microbial Horticulture, Department of Biosystems and Technology, Swedish University of Agricultural Sciences, P.O. Box 190, SE23422 Lomma, Sweden; sofia.windstam@gmail.com (S.W.); e.mulaosmanovic@hotmail.com (E.M.)

**Keywords:** abiotic conditions, challenge test, convenience food, cooling temperature, fresh-cut fruit, *Listeria monocytogenes*

## Abstract

Fresh-cut fruit salads (fruit cocktails) are marketed as a convenient food item with a limited shelf-life (4 days at 4 °C). Given rising electricity prices, increased cooling temperature during production, transport, and retail from 4 °C to 8 °C and extended shelf-life from four to eight days without compromising food safety are discussed. This study investigates the proliferation of *Listeria monocytogenes* in ready-to-eat (RTE) fresh-cut fruit cocktails at three temperature regimes. The fruit cocktail, consisting of pineapple, red apples, cantaloupe, and red grapes, was inoculated with a clinical strain of *L. monocytogenes* (SLV 444; CCUG 69007) and stored at 4 °C, 8 °C, or a dynamic temperature regime (4 °C for one day, 8 °C for seven days). After four-day storage at 4 °C, growth of *L. monocytogenes* was not supported. Despite the fruit cocktail’s pH below the minimum requirements of the target organism, all other treatments supported growth of *L. monocytogenes*, but below the legal limit of 2 log CFU + 1 g^−1^ per fruit cocktail. There is an increased risk of exceeding the microbiological safety end product criteria, especially at 8 °C or dynamic storage temperatures, if seemingly insignificant *Listeria* contamination is present in or on fruit cocktail ingredients.

## 1. Introduction

Fruits and vegetables are integral components of a healthy diet, offering a rich source of fiber, bioactive compounds, and essential minerals. Ready-to-eat (RTE) fresh-cut fruits, including single fruits and mixed fruit cocktails, have gained popularity due to their convenience and alignment with modern lifestyles [1,2] and support to reach a daily recommended intake of 500 g of fruits and vegetables. These products are commonly marketed in retail outlets, fast food chains, and institutional kitchens, where they undergo centralized preparation and distribution [3].

Although pre-cut fruits are often washed before preparation to remove visible contaminants, they are not subjected to further microbial decontamination methods (e.g., heat treatment or chemical disinfectants), making them susceptible to microbial contamination. A key concern is the presence of *Listeria monocytogenes*, a psychrotrophic bacterium that can thrive at low temperatures, posing significant food safety risks. Like other human pathogens, *L. monocytogenes* can proliferate in nutrient-rich fruit juices and on mechanically damaged tissue, such as the surfaces created during fruit slicing [4]. The rapid growth of *L. monocytogenes* on fresh-cut fruit can lead to spoilage and increase the risk of listeriosis, particularly for vulnerable populations, including the elderly, pregnant women, and immunocompromised individuals [5].

To mitigate these risks, maintaining a consistent cold chain throughout preparation, transport, and storage of fresh-cut fruit is critical. Refrigeration slows both microbial growth and fruit tissue degradation, thus prolonging shelf-life and reducing the likelihood of contamination [6,7]. Growth of *L. monocytogenes* is influenced by multiple factors, including the fruit type, ripeness, and the degree of mechanical damage [8,9]. Previous studies have shown that refrigeration at 4–5 °C significantly slows the growth of *Listeria* spp. and temperatures >8 °C may favor bacterial proliferation [10]. However, data on the interplay between temperature, fruit composition, and *Listeria* growth remains sparse, particularly for mixed fruit cocktails that incorporate multiple fruit types with varying susceptibilities to microbial contamination.

The present study explores the effect of different cooling regimes (4 °C; 8 °C; 4 °C during day 1 and 8 °C during the remaining time, hereafter called “dynamic (temperature) regime” or of “4 → 8 °C-regime”) on the growth of *L. monocytogenes* in industrially processed fresh-cut fruit cocktails, assessing how varying temperature scenarios influence its growth potential. *L. monocytogenes* was chosen as the model strain, as it naturally occurs on and in fruit and vegetables, survives and grows at low temperatures, and has caused outbreaks related to the consumption of cantaloupe [11] and apples [12]. The study was initiated by the fresh-cut processing industry and retailers concerned about rising energy costs and wondering about viable options for elevated temperature post-processing from 4 °C to 8 °C without compromising food safety. We hypothesized that (i) the an increase in storage temperature of fresh fruit cocktail from 4 °C to 8 °C significantly increases the occurrence of *L. monocytogenes* in fresh-cut fruit cocktails, (ii) that a middle ground agreement using a dynamic regime does not hamper the model organism’s proliferation, and (iii) that an extension of the recommended storage period can be extended from 4 days to 8 days supports the growth potential of *L. monocytogenes* in fresh-cut fruit cocktails.

## 2. Materials and Methods

### 2.1. Inoculum Preparation

A clinical strain of *L. monocytogenes* (SLV 444; CCUG 69007 available at the Culture Collection University of Gothenburg) labeled with spontaneous rifampicin resistance (200 µg mL^−1^) was used [13], and comparability in growth behavior with the wild type strain was confirmed. For inoculum preparation, the strain was subcultured twice: at 30 °C for 24 h, followed by low temperature growth at 8 °C for 36 h in Tryptone Soy Broth (TSB; BD Bacto Tryptic Soy Broth, Thermo Fisher, Waltham MA, USA) [13]. The cell culture was washed by repeated centrifugation (4 °C) and resuspension of the pellet in a 0.85% saline solution. The density of the stock solution was set to an optical density of OD_620_ 0.320 and then diluted, taking into account the expected fruit weight in the boxes (175 g). The total number of CFU of *L. monocytogenes* in each tray was Log 5.24 CFU, corresponding to Log 3 CFU g^−1^ fruit.

### 2.2. Experiment

The challenge study was conducted using film-sealed plastic PET trays containing 175 g of fresh-cut fruit cocktails (red apples, red grapes, cantaloupe, pineapple; no additives) packaged by a fresh-cut processing company directly before the onset of the experiment (Vidinge AB, Kävlinge, Sweden). Aliquots of 1 mL of inoculum were injected into each of the packages without breaking the package integrity. Control treatment fresh-cut fruit cocktails were mock inoculated with 1 mL of sterile NaCl (0.85%) [14]. The fresh-cut fruit cocktail packages with and without inoculation were placed at 4 °C and 8 °C and stored for eight days. The study was conducted with three temperature regimes, of which the first two follow the procedure of EURL guidance document [14] and the third one was suggested of special interest by the industry:

- Treatment 1: 4 °C for eight days;
- Treatment 2: 8 °C for eight days;
- Treatment 3: 4 °C for one day followed by 8 °C for the remaining storage time totaling eight days (dynamic temperature regime).

### 2.3. Analyses

The sampling schedule is presented in Table 1. For the analysis, the fruit cocktail from each box was divided for microbiological analysis, water activity analysis, and biochemical analysis. The controls were analyzed only on day 0 and day 8. For experimental reasons (infection control), pH was determined only on day 0 and day 8 in fruit taken from non-inoculated fruit cocktail packages [14].

#### 2.3.1. Microbiological Analyses

For the microbiological analysis, 25 g of fruit cocktail was weighed into filter bags and homogenized using a stomacher for 1 min at high speed (step 3). Samples were sedimented for 2 min. All liquid filtered through the filter bag membrane was then transferred into individual test tubes and centrifuged at 3200 rpm for 10 min at 4 °C. The supernatant was discarded, while the pellet was resuspended in 1 mL TRIS buffer [15] and then serially diluted in NaCl (0.85%) prior to plating dilutions on *L. monocytogenes* Harlequin™ *Listeria* Chromogenic Agar (Neogen, Lansing, MI, USA) supplemented with rifampicin (Merck KGaA, Darmstadt, Germany), conditions enabling selective growth of the bacterium. The general microbiota was assessed on TSB supplemented with 1.5% Bacto Agar by plating dilutions [13,14]. The presence of yeasts on yeast–malt agar was also analyzed ad hoc. Incubation temperature and duration are reported in Table 2. Samples were analyzed with three parallel replicates per fruit cocktail.

#### 2.3.2. Water Activity

Water activity (a_w_) was measured using the AquaLab Pre (Meter Group, Pullman, WA, USA), and water activity, temperature, and day point were recorded [13]. The instrument was calibrated before the first analysis for the day of analysis and after every 10 samples against two relevant standards (0.984; 1.000).

#### 2.3.3. Biochemical Analyses

Analyses of pH, titrable acidity, and total soluble solids were analyzed according to Ali et al. [16]. Briefly, pH and titrable acidity (TA) were determined using titration unit (Titroline easy; SCHOTT Instr. GmbH, Mainz, Germany); 100 mM NaOH to endpoint pH 8.3. TA values are shown as % citric acid *w*/*w*. Total soluble solids (TSS) were assessed using temperature-adjusted refractometer RFM 80 (Bellingham Stanley Ltd., Nottingham, UK).

#### 2.3.4. Calculation

Results on CFU were adjusted for weight and log-transformed (log_10_ + 1). The mean of each fruit cocktail was used for the statistical analysis. The growth parameters were calculated according to Equations (1)–(5) [14,17], namely for

• Number of generations (n).

(1) $$N=N_{0}{\;2}^{n}$$

With *N* expressing the final number of bacteria, *N*_0_ indicating the initial number of bacteria, and *n* depicting the number of generations.

• Generation time (g).

(2) $$g=\frac{t}{n}$$

With *g* indicating generation time, *t* expressing the time interval (h), and *n* depicting the number of generations.

• Specific growth rate (k).

(3) $$k=0.301\times \frac{t}{n}=\frac{0.301}{g}$$

With *g* indicating generation time, *t* expressing the time interval (h), and *n* depicting the number of generations and k for the specific growth rate, which corresponds to the slope of the curve.

• Division rate (*v*).

(4) $$v=\frac{1}{g}$$

With *v* and *g* expressing the division rate and number of generations, respectively.

• Growth potential (δ).

(5) $$\delta =\log N-\log N_{0}$$

With N indicating the final number of bacteria ((log CFU + 1) g^−1^ fruit) and *N*_0_ expressing the initial number of bacteria ((log CFU + 1) g^−1^ fruit)).

The experiment was conducted with six individual replicates per treatment and sampling event. Results were statistically analyzed using ANOVA (general linear model and one-way ANOVA, respectively) followed by Tukey’s test (*p* < 0.05) (Minitab version 19.20.20 and R version 4.2.2). The growth of *L. monocytogenes* (LogCFU + 1 (g fruit)^−1^) over all experimental days was analyzed using a one-way analysis of variance (ANOVA) to test the effect of temperature regime separately within each sampling day (dpi). For each time point, Tukey’s Honest Significant Difference (HSD) test was performed to determine pairwise differences among temperature treatments. Statistical analyses were conducted in R (version 4.2.2) using the aov() function for ANOVA and HSD.test() from the agricolae package for Tukey’s post hoc comparisons.

## 3. Results

### 3.1. Impact of Temperature Regime on L. monocytogenes

**Figure 1:**
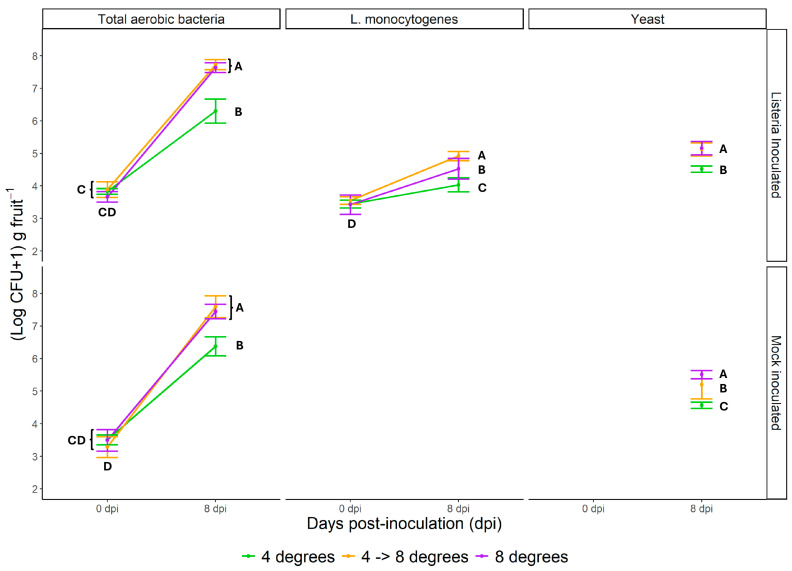
Viable counts ((Log CFU + 1) g fruit^−1^) of heterotrophic bacteria, *L. monocytogenes*, and yeasts when stored for eight days at 4 °C (green), 8 °C (purple), and 4 °C during day 1 and 8 °C during the remaining time (orange), respectively. Samples were taken at the beginning and end of the eight-day-long cooling period. The estimation of yeasts was performed ad hoc during the experiment and is therefore only presented for day 8. Data points indicate average values and standard deviations from six independent replicates at each sampling event. Different letters associated with the data points within the same group of organisms indicate significant differences according to general linear model and Tukey’s test (*p* < 0.05). For further statistical information, see Supplementary Tables S1 and S2.

**Figure 2:**
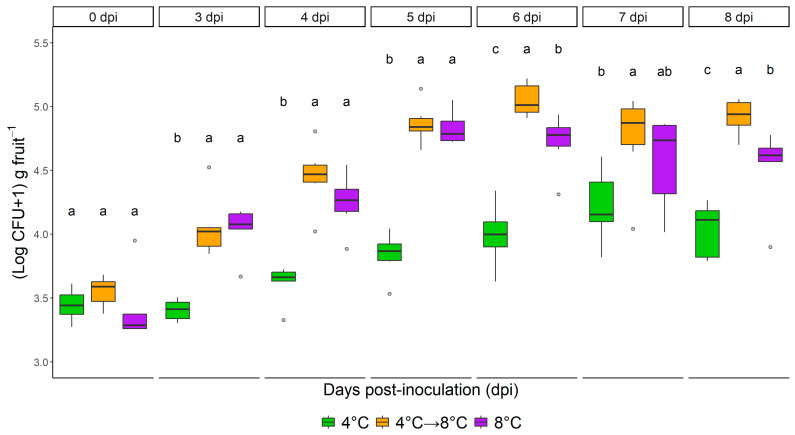
Proliferation of *Listeria monocytogenes* (((Log CFU + 1) g fruit^−1^), standard deviations) in fresh-cut fruit cocktails during eight days of storage at 4 °C (green) and 8 °C (purple) and one day at 4 °C and seven days at 8 °C (orange) (*n* = 6). The fresh-cut fruit cocktail consisted of red apples, red grapes, cantaloupe, and pineapple. Different letters above the bars within the sampling event indicate significant differences based on one-way ANOVA followed by Tukey’s test (*p* < 0.05).

After 8 days of storage, *L. monocytogenes* proliferated under all tested temperature regimes, with significantly slower growth at 4 °C compared to both continuous 8 °C and a dynamic regime (4 °C for 1 day followed by 8 °C for 7 days). At 4 °C, *L. monocytogenes* levels remained stable for up to 5 days and increased by less than 0.5 (log CFU + 1) g^−1^ by day 8. Notably, within the recommended 4-day shelf-life, no growth was observed at 4 °C, indicating limited risk under proper cold chain conditions (Figure 1 and Figure 2, Supplementary Tables S1 and S2).

Conversely, elevated temperatures supported growth, with increases of 1.37 (log CFU + 1) g^−1^ at 8 °C and 1.23 (log CFU + 1) g^−1^ under the dynamic regime. After 4 days, *L. monocytogenes* growth potential was higher in the dynamic treatment (0.99 (log CFU + 1) g^−1^) than at 8 °C (0.82 (log + 1 CFU) g^−1^), though variability among replicates (inter-package) under dynamic conditions was high. Significant differences in *Listeria* counts were evident from early time points (day 3 and 4), with both elevated regimes yielding >10-fold increases by day 8.

Temperature strongly influenced growth kinetics (Table 3): The doubling time at 4 °C was 13.68 h, compared to 5.42 h at 8 °C and 4.39 h under dynamic conditions, thus 2.5 and >3 times faster at abuse temperature regime. Dynamic temperature exposure notably accelerated *L. monocytogenes* proliferation relative to constant 8 °C, underscoring the risk posed by temperature abuse during storage and distribution.

### 3.2. Impact of Temperature Regime on Heterotrophic Organisms

Heterotrophic bacteria were analyzed only on day 0 (immediately after the start of the experiment) and day 8 (at the end of the experiment) in both inoculated and uninoculated fruit cocktails (Figure 1). Significant growth of heterotrophic bacteria was detected in all treatments (Figure 1, Table 3). Growth was significantly lower in fresh-cut fruit cocktails stored at 4 °C than when exposed to 8 °C and the dynamic temperature regime. The incidence of heterotrophs was slightly, but not significantly, lower in fresh-cut fruit cocktails inoculated with *L. monocytogenes*. Also, for heterotrophic bacteria, less time was needed for doubling when the sealed trays were stored at 8 °C compared to continuous regime at 4 °C. The difference was not as striking as for *L. monocytogenes*. The rate of multiplication was slower in the presence of *L. monocytogenes*.

The presence of yeasts was therefore evaluated ad hoc on day 8, and the results are shown in Figure 1. Significant differences in yeast incidence were found between fresh-cut fruit cocktails stored continuously at 4 °C and the other two temperature regimes. The occurrence of yeasts was highest when fresh-cut fruit cocktails had been stored continuously at 8 °C. Significant differences between the dynamic and continuous 8 °C treatments were only observed in fresh-cut fruit cocktails free from *L. monocytogenes*. When yeast levels were compared between fresh-cut fruit cocktails with and without the addition of *L. monocytogenes*, a trend towards lower yeast levels were observed in the presence of *Listeria*.

### 3.3. Abiotic Factors

Abiotic factors (pH, water activity, titrable acidity, and total soluble solids) essential for the growth of *L. monocytogenes* are presented in Table 4. No significant differences were observed for pH and water activity for any of the temperature regimes between the two readings. Total soluble solid values decreased in all regimes between the start and end of the experiment, but significant differences were only observed at a constant or dynamic storage temperature of 8 °C.

## 4. Discussion

Refrigeration is essential for extending the shelf-life of fresh-cut fruit cocktails by slowing both natural degradation and microbial growth. Fruit tissue degradation and microbial spoilage are closely linked. The processing-induced (e.g., slicing, dicing) “wounds” release juices and increase surface area, creating favorable conditions for microbial proliferation [2,18] including pathogens such as *L. monocytogenes* and spoilage microorganisms. Given its real-world scenario, the present challenge study provides a novel perspective relevant for the processing industry but also for distribution, retailers and consumers.

*L. monocytogenes*, a psychrotrophic bacterium capable of multiplying at temperatures as low as −1.5 °C [14], can rapidly colonize such fruit tissue. This coupled with its facultative anaerobic nature and tolerance to high water activity and salt concentrations, makes it particularly challenging to control in refrigerated fruit products [4,19]. Various studies have considered the fate of *L. monocytogenes* in single fresh-cut fruit items (e.g., apple [20,21,22], cantaloupe [20,22,23,24,25,26,27,28,29], grape [20], mango [27], papaya [30,31], pear [30], pineapple [20,30], and watermelon [30,31]), whereas challenge studies using fresh-cut fruit cocktails are rare. The fruit cocktail used in this study consisted of a mix of red apples, red grapes, pineapple, and cantaloupe (without additives), a matrix dense in organic nutrients but heterogenous in pH as well as the porosity and surface density of the cut surfaces, thus providing microenvironments permissive to proliferation of the target organism. This heterogenicity in matrix should be taken into account when considering intra-package variations.

This study set out to test three primary hypotheses regarding the behavior of *L. monocytogenes* in fresh-cut fruit cocktails: (i) that increasing storage temperature from 4 °C to 8 °C significantly enhances pathogen proliferation; (ii) that a dynamic storage regime—initial cold storage followed by moderate temperature elevation—permits similar growth; and (iii) that extending the storage period from four to eight days supports pathogen development. The findings offer robust support for all three hypotheses and provide mechanistic insight into how subtle deviations in refrigeration parameters can affect microbial safety in RTE fruit matrices. Our study employed a cold-adapted inoculum grown under nutrient-rich conditions [25,32] in order to mimic real-world conditions where *L. monocytogenes* is pre-exposed to environmental stressors before contaminating produce. It is worthwhile to note that the present study is based on one fresh-cut fruit cocktail mix and one clinical *L. monocytogenes* strain and needs to be verified in provocation studies involving a diversity of fruit cocktail mixes and additional strains [33].

### 4.1. Effect of Temperature Increase (4 °C to 8 °C)

Our results confirm that *L. monocytogenes* does not proliferate under strict refrigeration at 4 °C within a four-day period. This aligns with established literature indicating that low temperatures impose significant metabolic constraints on the organism [20,30]. However, measurable growth occurred at 8 °C—a temperature frequently encountered during distribution or in domestic refrigerators—validating our first hypothesis. Reaching levels above the 2 log CFU g^−1^ threshold within 3–4 days of storage at 8 °C suggests that even slight increases in temperature can accelerate microbial risks, particularly for vulnerable populations such as the elderly and immunocompromised individuals [5]. Previous studies similarly reported log-scale increases in *Listeria* populations at mild temperature abuse conditions, including on fresh-cut cantaloupe and mixed fruit matrices [25,26,29].

### 4.2. The Impact of Dynamic Temperature Regime

We also examined a simulated middle-ground approach involving dynamic storage (4 °C for 24 h followed by 7 days at 8 °C). This treatment supported *L. monocytogenes* proliferation to the same extent as constant 8 °C exposure, confirming the second hypothesis. Notably, even short initial refrigeration did not prevent growth once temperature increased. This scenario is realistic for modern cold chains where initial compliance is often followed by later-stage lapses during retail or consumer handling [34,35]. Our results clearly state that such a temperature regime by the processing industry viewed as a compromise to please energy cost concerns during distribution and retail adventures food safety. This has also been demonstrated in provocations using exclusively cantaloupe, where similar growth curves were presented [20].

Given its psychrotrophic nature, *L. monocytogenes* is able to survive low-temperature conditions and resume growth when suitable conditions are re-established. These findings mirror previous experimental results showing the target organism’s ability to recover and proliferate after intermittent exposure to temperature abuse, including brief exposures as short as four hours at 8–20 °C [24,28].

### 4.3. The Effect of Shelf-Life Extension (From Four to Eight Days of Storage)

Extending the shelf-life from four to eight days facilitated further pathogen development under both constant and dynamic temperature regimes, supporting our third hypothesis. Although population levels remained below the EU safety threshold of 2 log CFU g^−1^ [36], the risk of exceeding these limits under less controlled real-world scenarios is substantial. Other studies have shown similar trends, where *Listeria* reached critical levels over 7–15 days in cantaloupe and mixed fruit samples, even under refrigeration [23,26,37].

These results reinforce the principle that the duration of storage time is an enabling factor for *L. monocytogenes* proliferation—especially when coupled with permissive environmental conditions. For industry stakeholders considering shelf-life extensions, this highlights the importance of verifying that time-based decisions do not compromise microbiological safety margins. The findings are also important for consumers and storage in kitchen refrigerators with oscillating cooling conditions, and prolonged storage may be supportive for the growth of *L. monocytogenes*.

### 4.4. Growth Mechanisms Under Adverse Conditions

While the fruit cocktail exhibited an average pH below the known minimum for *L. monocytogenes* growth, we nonetheless observed measurable proliferation. This paradox might be explained by known acid tolerance mechanisms, nutrient release from damaged fruit tissues, and the protective effect of food matrices [38]. It is tempting to speculate that cantaloupe was implicated as a high-risk component in the studied fruit cocktail matrix, as it was cited in multiple studies with respect to log increases exceeding 3.0 within one week at abusive temperatures [24,25].

These findings also underscore the limitations of pH as a standalone safety metric. The presence of spatial heterogeneity, localized buffering, and inter-fruit interactions in mixed salads means that *Listeria* can exploit microenvironments despite globally inhospitable conditions.

### 4.5. Microbial Behavior and Food Safety Concerns

Although not explicitly hypothesized, this challenge study also followed up on the heterotrophic microbiota, demonstrating a clear temperature-dependent response in heterotrophic bacterial proliferation within fresh-cut fruit cocktails and supporting prior findings on spoilage organisms [39,40,41]. These results, beautifully illustrated by the smell and package metamorphosis, reinforce the importance of strict cold chain control in limiting microbial growth in minimally processed fruit products. Despite the challenges posed by *L. monocytogenes*, it should be noted that outbreaks related to fruit products remain rare. For example, the incidence of *L. monocytogenes* in fruit was found to be just 0.51% in a Canadian study [42], and in Switzerland, no *L. monocytogenes* was detected in 64 samples of fresh-cut fruit [43]. Nevertheless, although log 2 proliferation was not exceeded in any of the treatments, our findings demonstrate how narrow the safety margin is under less-than-optimal conditions. This, in combination with the repeated isolation of *L. monocytogenes* from different zones in fresh-cut processing facilities highlights that the problem per se may not be ignored [44]. Also, the severity of listeriosis, which can result in severe outcomes such as meningitis and sepsis in vulnerable populations, remains a significant public health concern [14,34]. The high mortality rate and the potential for transmission to pregnant women highlight the importance of maintaining strict food safety protocols, particularly for high-risk consumers.

Thus, food safety policies should go beyond endpoint testing and incorporate proactive risk mitigation strategies. These include real-time temperature monitoring, conservative shelf-life determination, consumer education, and the application of hurdle technologies. Regulatory reliance solely on compliance with fixed microbial limits may provide false reassurance if cold chain integrity is not maintained throughout the product life cycle.

## 5. Conclusions and Practical Implications

In conclusion, the present study validates all three hypotheses. While refrigeration effectively slows down both microbial growth and fruit degradation, the present study highlights that increasing the storage temperature of fresh-cut fruit salads from 4 °C to 8 °C and extending storage time from 4 to 8 days is not advisable without further risk mitigation strategies [45]. The increase in *L. monocytogenes* growth at 8 °C, combined with the rapid proliferation of yeasts that degrade sensory quality, suggests that maintaining the recommended storage temperature of 4 °C is essential for ensuring both microbial safety and consumer acceptance. Any efforts to extend shelf-life must carefully consider the trade-offs between food safety, fruit quality, and consumer perceptions. There is an increased risk of exceeding the microbiological safety end product criteria of 100 CFU g^−1^ food item, in particular if using 8 °C as maximum storage temperatures or dynamic storage temperatures. Even for storage temperatures at 4 °C, there is a risk that the safety will be compromised. The content of *L. monocytogenes* at day 0 is crucial for the storage temperatures.

This study employed a controlled laboratory model using a single clinical strain, which, while useful for baseline risk evaluation, may not fully reflect the diversity of strains encountered in food systems. Future work should evaluate strain mixtures and other fresh-cut fruit cocktail compositions, simulate retail and consumer handling, and test the effect of protective atmospheres, pH modifiers, or natural antimicrobials in fresh-cut matrices. Additionally, exploring fruit cocktails without cantaloupe may help identify lower-risk alternatives for RTE applications. More holistic risk assessments should also consider the role of native microbiota, cross-contamination during preparation, and post-purchase handling variability.

## Figures and Tables

**Table 1:** Sampling schedule for *Listeria monocytogenes*, the heterotrophic microbiota, yeasts, and abiotic parameters (pH, water activity (a_w_), total soluble solids, and titrable acidity).

Parameter	Day						
	0	3	4	5	6	7	8
*Listeria monocytogenes*	•	•	•	•	•	•	•
Heterotrophic microbiota	•						•
Yeasts							•
pH	•						•
Total soluble solids	•						•
Titrable acidity	•						•
Water activity	•	•	•	•	•	•	•

**Table 2:** Incubation conditions (temperature, °C; length, h) for heterotrophic bacteria using Tryptic Soy Agar (TSA), *Listeria monocytogenes* (Harlequin Agar), and yeasts (yeast malt agar).

Microbiological Medium	Incubation	
	Temperature	Length
Tryptic Soy Agar	25	48
Harlequin Agar	37	24
Yeast malt Agar	25	72

**Table 3:** Growth parameters of *L. monocytogenes* and heterotrophic bacteria in fresh-cut fruit cocktail during storage for 8 days at 4 °C, 8 °C, and with dynamic temperature control (day 1: 4 °C; remaining seven days: 8 °C). The number of generations (n), generation time (*g*; h/generation), specific growth rate (*k*; number of generations/h), and division rate (*v*, reciprocal to generation time *g*) were calculated based on viable count data shown in Figure 1 and Figure 2.

Temperature Regime	*n*	*g*	*k*	*v*
*L. monocytogenes*				
4 °C	1.96	10.20	0.03	0.10
4 °C → 8 °C	4.55	4.39	0.07	0.23
8 °C	3.69	5.42	0.06	0.18
Heterotrophic bacteria in non-inoculated fresh-cut fruit cocktails				
4 °C	9.5	2.11	0.14	0.48
4 °C → 8 °C	14.28	1.40	0.22	0.71
8 °C	13.15	1.52	0.20	0.66
Heterotrophic bacteria in *L. monocytogenes*-inoculated fresh-cut fruit cocktails				
4 °C	8.21	2.44	0.12	0.41
4 °C → 8 °C	12.79	1.56	0.19	0.64
8 °C	13.22	1.51	0.20	0.66

**Table 4:** Abiotic factors (pH; water activity, a_w_; total acidity, %; total soluble solids, %) in fresh-cut fruit cocktails at the start (day 0) and end (day 8) of the study. Fresh-cut fruit cocktails were stored continuously at 4 °C and 8 °C for eight days, respectively, or in dynamic temperature control with 4 °C on day 1 and 8 °C for the remaining seven days. (*n* = 6).

Parameter	4 °C		4 °C → 8 °C		8 °C	
	Day 0	Day 8	Day 0	Day 8	Day 0	Day 8
pH	3.85 ± 0.19 A ^1^	3.91 ± 0.22 A	3.89 ± 0.06 A	3.95 ± 0.09 A	3.96 ±0.08 A	3.92 ± 0.10 A
a_w_	0.982 ± 0.003 A	0.985 ± 0.004 A	0.980 ± 0.002 A	0.980 ± 0.003 A	0.980 ± 0.002 A	0.980 ± 0.003 A
Titrable acidity	0.56 ± 0.06 A	0.54 ± 0.08 A	0.55 ± 0.02 B	0.63 ± 0.05 A	0.60 ± 0.04 A	0.62 ± 0.06 A
Total soluble solids	14.14 ± 0.64 A	13.70 ± 0.72 A	14.09 ± 0.27 A	13.26 ± 0.54 B	14.46 ± 0.55 A	13.70 ± 0.55 B

^1^ Values within the same row and storage temperature accompanied by different letters differ significantly according to one-way ANOVA and Tukey test (*p* < 0.05).

## Data Availability

The datasets generated during and analyzed during the current study are available at https://doi.org/10.5878/3c62-ps41.

## Supplementary Materials

The following supporting information can be downloaded at https://www.mdpi.com/article/10.3390/foods14203523/s1, Table S1: The analysis of variance (general linear model) on data recruited from the provocation experiment on the growth of *Listeria monocytogenes* in fresh-cut fruit cocktails consisting of red apples, red grapes, pineapple, and cantaloupe without additives; 175 g boxes with fresh-cut fruit cocktails were inoculated with either *L. monocytogenes* or 0.85 NaCl and kept for 8 days at either 4 °C or 8 °C or at 4 °C for the first day and 8 °C for the remaining time (“dynamic temperature regime”). The statistical analysis considers values obtained for *Listeria monocytogenes*, the heterotrophic microbiota, directly after inoculation and after 8 days storage. Yeasts and molds were only assessed after 8 days of storage. Table S2: Descriptive statistics, the two-sample T-test, confidence intervals, and probability levels on data recruited from the provocation experiment on growth of *Listeria monocytogenes* in fresh-cut fruit cocktails consisting of red apples, red grapes, pineapple, and cantaloupe without additives; 175 g boxes with fresh-cut fruit cocktails were inoculated with either *L. monocytogenes* or 0.85 NaCl and kept for 8 days at temperature regimes (TR) of either 4 °C or 8 °C or at 4 °C for the first day and 8 °C for the remaining time (“dynamic temperature regime”; 4 → 8). The statistical analysis considers values obtained for *Listeria monocytogenes*.
