# Supplementary material for: Chill or Thrill? The Effect of Storage Temperature Regime on Listeria Growth in Fresh-Cut Fruit Cocktails
Source: Foods. 2025 Oct 16;14(20):3523. doi: 10.3390/foods14203523 (PMC12563481; doi:10.3390/foods14203523)
Supplement: Supplementary file 1 [file foods-14-03523-s001.zip › foods-3855864-supplementary.pdf]

Supplementary Materials

Supplementary Table S1. Analysis of variance (general linear model) on data recruited from the provocation experiment on growth of *Listeria monocytogenes* in fresh-cut fruit cocktails consisting of red apples, red grapes, pineapple and cantaloupe without additives. 175 g boxes with fresh-cut fruit cocktails were inoculated with either *L. monocytogenes* or 0.85 NaCl and kept for 8 days at either 4 °C or 8 °C or at 4 °C for during the first day and 8 °C during the remaining time ("dynamic temperature regime"). The statistical analysis considers values obtained for Listeria monocytogenes the heterotrophic microbiota directly after inoculation and after 8 days storage. Yeasts and moulds were only assessed after 8 days of storage.

**Source**	**DF**	**Adj SS**	**Adj MS**	**F-Value**	**P-Value**
Listeria monocytogenes					
Temperature regime (TR)	2	1.4139	0.70695	15.38	0.000
Day post inoculation (DPI)	1	8.7774	8.77740	190.95	0.000
TR*DPI	2	0.8870	0.44352	9.65	0.001
Error	28	1.2871	0.04597		
Total	33	12.7491			
Heterotrophic microbiota, non-inoculated					
TR	2	2.117	1.058	13.18	0.000
DPI	1	123.823	123.823	1541.74	0.000
TR*DPI	2	3.378	1.689	21.03	0.000
Error	30	2.409	0.080		
Total	35	131.727			
Heterotrophic microbiota, inoculated					
TR	2	3.671	1.835	40.46	0.000
DPI	1	105.774	105.774	2331.68	0.000
TR*DPI	2	4.189	2.095	46.17	0.000
Error	30	1.361	0.045		
Total	35	114.996			
Yeast and moulds, non-inoculated					
TR	2	2.759	1.37964	19.17	0.000
Error	15	1.080	0.07197		
Total	17	3.839			
Yeast and moulds, inoculated					
TR	2	1.5640	0.78198	25.31	0.000
Error	15	0.4635	0.03090		
Total	17	2.0274			

Supplementary Table S2. Descriptive statistics, two sample T-test, confidence intervals as well as probability levels on data recruited from the provocation experiment on growth of *Listeria monocytogenes* in fresh-cut fruit cocktails consisting of red apples, red grapes, pineapple and cantaloupe without additives. 175 g boxes with fresh-cut fruit cocktails were inoculated with either *L. monocytogenes* or 0.85 NaCl and kept for 8 days at temperature regimes (TR) of either 4 °C or 8 °C or at 4 °C for during the first day and 8 °C during the remaining time (“dynamic temperature regime”; 4 →8). The statistical analysis considers values obtained for *Listeria monocytogenes.*

TR	Day	N	Mean	StDev	SE Mean	Diffe-rence	95% CI for	T-Value	DF	P-Value
4	0	6	3.444	0.123	0.050	-0.59	(-0.856, -0.323)	-5.41	6	0.002
	8	5	4.034	0.216	0.097					
4 →8	0	6	3.551	0.118	0.048	-1.369	(-1.5386, -1.1993)	-18.25	9	<0.001
	8	6	4.92	0.141	0.057					
8	0	5	3.425	0.297	0.1300	-1.101	(-1.529, -0.673)	-5.94	8	<0.001
	8	6	4.526	0.317	0.1300					
